# Compound Kushen Injection Induces Immediate Hypersensitivity Reaction Through Promoting the Production of Platelet-Activating Factor via *de Novo* Pathway

**DOI:** 10.3389/fphar.2021.768643

**Published:** 2021-10-08

**Authors:** Yuan Gao, Lina Hai, Yuan Kang, Wenjie Qin, Fang Liu, Runlan Cai, Xiuwei Yang, Yun Qi

**Affiliations:** ^1^ Institute of Medicinal Plant Development, Chinese Academy of Medical Sciences and Peking Union Medical College, Beijing, China; ^2^ Academy for Advanced Interdisciplinary Studies, Peking University, Beijing, China; ^3^ Beijing Zhendong Guangming Pharmaceutical Research Institute, Beijing, China; ^4^ State Key Laboratory of Natural and Biomimetic Drugs, Department of Natural Medicines, School of Pharmaceutical Sciences, Peking University, Beijing, China

**Keywords:** compound kushen injection, matrine, platelet-activating factor, non-immunologic immediate hypersensitivity reaction, *de novo* pathway of platelet-activating factor

## Abstract

Compound Kushen Injection (CKI) is a *bis-*herbal formulation extracted from Kushen (Radix Sophorae Flavescentis) and Baituling (Rhizoma Heterosmilacis Yunnanensis). Clinically, it is used as the adjuvant treatment of cancer. However, with the increased application, the cases of immediate hypersensitivity reactions (IHRs) also gradually rise. In this study, we investigated the underlying mechanism(s) and active constituent(s) for CKI-induced IHRs in experimental models. The obtained results showed that CKI did not elevate serum total IgE (tIgE) and mouse mast cell protease 1 (MMCP1) after consecutive immunization for 5 weeks, but could induce Evans blue extravasation (local) and cause obvious hypothermia (systemic) after a single injection. Further study showed that alkaloids in Kushen, especially matrine, were responsible for CKI-induced IHRs. Mechanism study showed that various platelet-activating factor (PAF) receptor antagonists could significantly counter CKI-induced IHRs locally or systemically. In cell system, CKI was able to promote PAF production in a non-cell-selective manner. In cell lysate, the effect of CKI on PAF production became stronger and could be abolished by blocking *de novo* pathway. In conclusion, our study identifies, for the first time, that CKI is a PAF inducer. It causes non-immunologic IHRs, rather than IgE-dependent IHRs, by promoting PAF production through *de novo* pathway. Alkaloids in Kushen, especially matrine, are the prime culprits for IHRs. Our findings may provide a potential approach for preventing and treating CKI-induced IHRs.

## Introduction

Compound Kushen Injection (CKI), also called “fufangkushen injection”, is a Chinese patent medicine extracted from two herbs: Kushen (Radix Sophorae Flavescentis) and Baituling (Rhizoma Heterosmilacis Yunnanensis). It was approved by State Food and Drug Administration of China for the adjuvant treatment of cancer more than 20 years ago (Guo et al., 2015). Clinically, CKI is widely used for moderating pain and reducing side effects in combination with conventional analgesics, chemotherapy or radiotherapy, thus improving the life quality of patients (Yanju et al., 2014; Qi et al., 2015). However, with the increased application, adverse drug reactions (ADRs) were also reported. The clinical manifestations of CKI-induced ADRs involved in many systems (e.g., gastrointestinal, cutaneous, nervous, cardiovascular, respiratory, etc.), of which approximately 70% of ADRs occurred within 30 min after the first injection of CKI, belonging to typical immediate hypersensitivity reactions (IHRs) (Dian et al., 2020).

IHRs can be triggered by a series of vasoactive mediators (e.g., complement-derived anaphylatoxin, histamine, platelet-activating factor (PAF), leukotriene, and bradykinin, etc.) that increase vascular permeability, induce hypothermia, or cause smooth muscle contraction, etc. (Golden, 2004; Simons, 2010; Muñoz-Cano et al., 2016). Drugs, foods, and insect stings are the most common causes of IHRs (Decker et al., 2008). Drug-induced IHRs are a subgroup of unexpected ADRs initiated by drug-exposure at a dose tolerated by normal individuals and characterized by urticaria, angioedema, bronchospasm, and anaphylaxis, etc. (Demoly et al., 2014). Since IHRs may occur by immune-mediated or nonimmune-mediated mechanisms, they are classified as immunologic IHRs (IN-IHRs) or non-immunologic IHRs (NIN-IHRs). Regarding the former, most drugs act as haptens or prohaptens to become immunogenic thus inducing IN-IHRs. Although IgG-mediated IHRs were also reported in animal models (Muñoz-Cano et al., 2016), IN-IHRs are almost always mediated by IgE in humans (Jimenez-Rodriguez et al., 2018). Following secondary drug-exposure, the antigen, presumably hapten-protein complex, cross-links specific IgE that has bound to the high-affinity IgE receptor (FcεRI) on cytomembrane, and consequently stimulates the release of preformed mediators or *de novo* synthesized ones (Ishizaka et al., 1972). NIN-IHRs cannot be distinguishable from IgE-mediated IHRs clinically (Aun et al., 2017) and have also been described, such as direct degranulation of mast cells/basophils (Johansson et al., 2004), cytokine-release reactions (Nakamura and Murata, 2018), complement activation (Gao et al., 2018; Gao et al., 2020a), or contact system activation (Gao et al., 2020b), etc.

Although CKI-induced IHRs had been widely reported, no previous study focused on this. In the present study, we systemically investigated the underlying mechanism(s) and active constituent(s) for CKI-induced IHRs in experimental models. Our findings may provide a potential strategy for preventing and treating CKI-caused IHRs.

## Methods

### Materials and Reagents

CKI, *Sophora flavescens* Ait. (SF)-free CKI, *Heterosmilax yunnanensis* Gagnep. (HY)-free CKI, and macrozamin (CAS^#^6327-93-1) were prepared by Beijing Zhendong Guangming Pharmaceutical Research Institute (Beijing, China) and authenticated by the quality controller Qin Li. Compound 48/80 (C48/80, Cat^#^C2313), propranolol, triprolidine and Evans blue were from Sigma-Aldrich (St Louis, MO, United States). Cimetidine and ginkgolide B were from Tokyo Chemical Industry (Tokyo, Japan). Metergoline, icatibant, rupatadine fumarate and meclofenoxate (Mecl) were from MedChemExpress (Monmouth Junction, NJ, United States). TSI-01 was from GLPBIO Co. (Montclair, CA, United States). SB290157 and PMX53 were obtained from Santa Cruz Biotechnology (Santa Cruz, CA, United States) and Tocris Bioscience (Bristol, United Kingdom), respectively. MoTP was from Abcam (Cambridge, United Kingdom). ELISA kits for mouse total IgE (tIgE) and mouse mast cell protease 1 (MMCP1) were from Biolegend (San Diego, CA, United States) and Invitrogen (San Diego, CA, United States), respectively. PAF ELISA kit was from BioVision Incorporated (Milpitas, CA, United States). Imject™ aluminium adjuvant was obtained from Thermo Scientific (Waltham, MA, United States). Matrine (CAS^#^519-02-8) and oxymatrine (CAS^#^16837-52-8) were from National Institutes for Food and Drug Control (Beijing, China). Sophocarpine (CAS^#^145572-44-7), sophoridine (CAS^#^6882-68-4) and oxysophocarpine (CAS^#^26904-64-3) were from Chengdu Herbpurify Co., LTD (Chengdu, China). Piscidic acid (CAS^#^469-65-8) and trifolirhizin (CAS^#^6807-83-6) were obtained from Institute of Chinese Materia Medica China Academy of Chinese Medical Sciences (Beijing, China) and Guangzhou Langou biotech Co. LTD (Guangzhou, Guangdong, China), respectively. Shrimp tropomyosin (ST) was extracted from *Penaeus japonicus* and purified using an isoelectric precipitation method as we previously described (Gao et al., 2017). All other reagents were of analytical grade.

### Cells and Animals

Rat basophilic leukemia cell line (RBL-2H3) and human monocytic cell line (THP-1) were obtained from Center for Excellence in Molecular Cell Science of Chinese Academy of Sciences (Shanghai, China). Human mast cell line LAD2 (Columbia University, United States) was presented by Prof. Renshan Sun (the Third Military Medical University, Chongqing, China). Whole blood cells were obtained from BALB/c mice (female, 18–20 g) which were from Vital River Experimental Animal Services (Beijing, China) and housed in a vivarium under standard conditions of temperature and humidity and with a 12 h light/dark cycle.

### Ethics Statement

All animal care and experimental protocols and procedures were approved by the Committee for Care and Welfare of Laboratory Animals in Institute of Medicinal Plant Development of Chinese Academy of Medical Sciences and Peking Union Medical College. Animal experiments were reported in compliance with the ARRIVE guidelines. Anesthetic drug (isoflurane) and all other necessary measures were used to reduce animals suffering during experimental procedures.

### HPLC Analysis

The constituents in CKI, SF-free CKI and HY-free CKI were assayed by a Waters HPLC system. A high resolution HPLC column (Waters XSelect CSHTM C18, 5 μm, 4.6 mm × 250 mm) was used. Column temperature was set at 30°C. Mobile phases were 0.2% (v/v) KH_2_PO_4_ in water (pH 3.0) and CH_3_OH with a gradient elution as listed in Table 1. The flow rate was 0.6 ml/min and the detection wavelength was 211 nm.

**TABLE 1:** Mobile phase condition of chromatographic separation.

min	CH_3_OH (%)	0.2% KH_2_PO_4_ (%)
0–10	3	97
10–15	3–5	97–95
15–24	5–15	95–85
24–30	15	85
30–55	15–85	85–15
55–60	85	15
60–75	3	97

### Measurement of tIgE and MMCP1 in Serum

To amplify Th2 response, aluminium adjuvant was used during mouse immunization. The mice were intraperitoneally injected with ST (60 µg/mouse) or CKI (50 µl/mouse) or equivoluminal normal saline (NS) in the presence of aluminum adjuvant (100 µl/mouse) once a week. Seven days after the fifth immunization, serum tIgE and MMCP1 were determined using the commercial ELISA kits. ST was used as a positive control.

### Evans Blue Extravasation Assay

Evans blue extravasation in mouse hind paw was measured as previously described (McNeil et al., 2015). Briefly, the mice were injected (i.v.) with Evans blue (0.65 μmol). Five minutes later, one paw was intraplantarly injected with CKI (10 μl) or C48/80 (0.3 μg/paw), and the other paw was injected with 10 μl of NS. Thirty minutes later, the mice were euthanized. Paw tissues were collected and Evans blue in the paw tissues was extracted by DMF (CAS^#^68-12-2) at 50°C overnight (>20 h). Optical density (OD) values were read at 620 nm. C48/80 was used as a positive control. For local antagonist experiment, 10 μl of antagonist or NS was intraplantarly injected into the paw 10 min before Evans blue injection. For systemic antagonist experiment, the mice were intravenously injected with PAF receptor antagonist or NS 30 min before Evans blue injection.

### Anaphylactic Shock Assay

To increase the severity of anaphylaxis, the mice were pretreated (i.v.) with propranolol (0.118 μmol/mouse) (TenBrook et al., 2004; Khodoun et al., 2009) 20 min before CKI (1.25–5 ml/kg) or C48/80 (4 mg/kg) challenge (i.p.). The mice in the negative control group were received equivoluminal NS. Thirty minutes later, the rectal temperature was measured (Gao et al., 2021).

### Measurement of PAF

Cells were incubated with test substance at 37°C for 1 h. The cell culture supernatant was collected and centrifuged (1,000 × g) at 4°C for 20 min. The supernatant was used for PAF assay by using commercial PAF ELISA kit. PAF concentration was calculated according to the standard curve. In the cell-free experiment, RBL-2H3 cells were lysed on ice by ultrasonication and the obtained cell lysates were centrifuged (5,000 × g) at 4°C for 5 min. The supernatant (85 μg protein, 80 μl/well) was incubated with CKI or matrine at 37°C for 1 h in the presence or absence of blocker. PAF level was determined according to the above description.

### Data Presentation

The data were reported as the mean ± SD from a representative experiment. All of the experiments reported in this work were repeated at least three times with the same pattern of results. All data were analyzed by GraphPad Prism 8.0 using one-way ANOVA followed by the Tukey posttest. A student’s *t*-test was used when only two groups were compared. *p* < 0.05 was considered significant.

## Results

### Fingerprints and Constituent Identification

**FIGURE 1:**
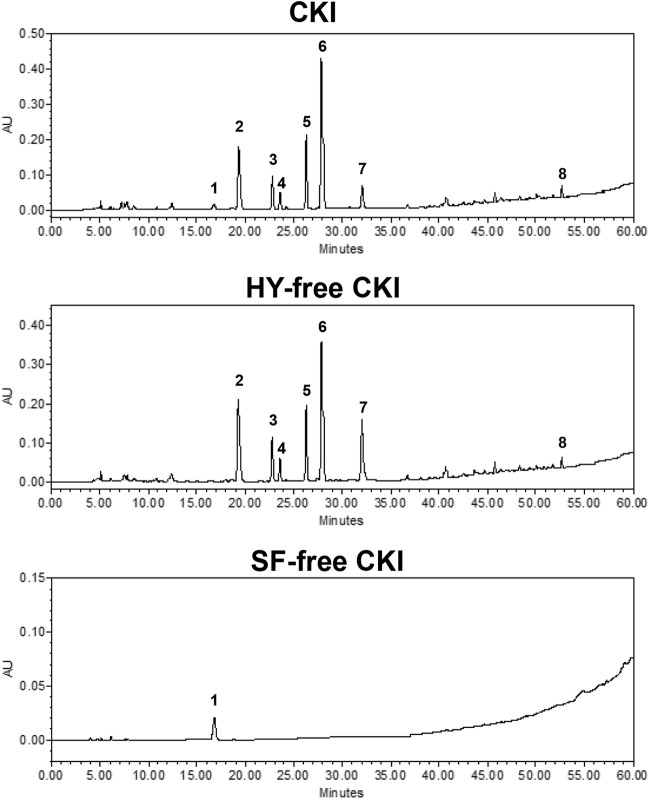
Chromatogram fingerprints of CKI, HY-free CKI and SF-free CKI. 1. macrozamin (CAS^#^6327-93-1); 2. matrine (CAS^#^519-02-8); 3. sophocarpine (CAS^#^145572-44-7); 4. sophoridine (CAS^#^6882-68-4); 5. oxysophocarpine (CAS^#^26904-64-3); 6. oxymatrine (CAS^#^16837-52-8); 7. piscidic acid (CAS^#^469-65-8); 8. trifolirhizin (CAS^#^6807-83-6). CKI, Compound Kushen Injection; HY, *Heterosmilax yunnanensis* Gagnep.; SF, *Sophora flavescens* Ait.

The main constituent profiles of CKI, HY-free CKI and SF-free CKI were analyzed by HPLC-UV, respectively. The retention time values of the identified constituents were compared with that of the reference substances. Eight constituents (macrozamin, matrine, sophocarpine, sophoridine, oxysophocarpine, oxymatrine, piscidic acid, trifolirhizin) were identified (Figure 1), and six of them were quantified (Table 2).

**TABLE 2:** Concentrations of six constituents in CKI.

Constituent	Concentration (mM)		
	CKI	HY-free CKI	SF-free CKI
macrozamin	1.38	—	2.79
matrine	16.99	29.26	—
sophocarpine	4.51	7.80	—
sophoridine	3.58	5.93	—
oxysophocarpine	9.00	11.83	—
oxymatrine	31.66	34.59	—

CKI, Compound Kushen Injection; HY, *Heterosmilax yunnanensis* Gagnep.; SF, *Sophora flavescens* Ait.

### CKI-Induced IHR Is Independent of IgE/FcεRI-Mediated Signaling

**FIGURE 2:**
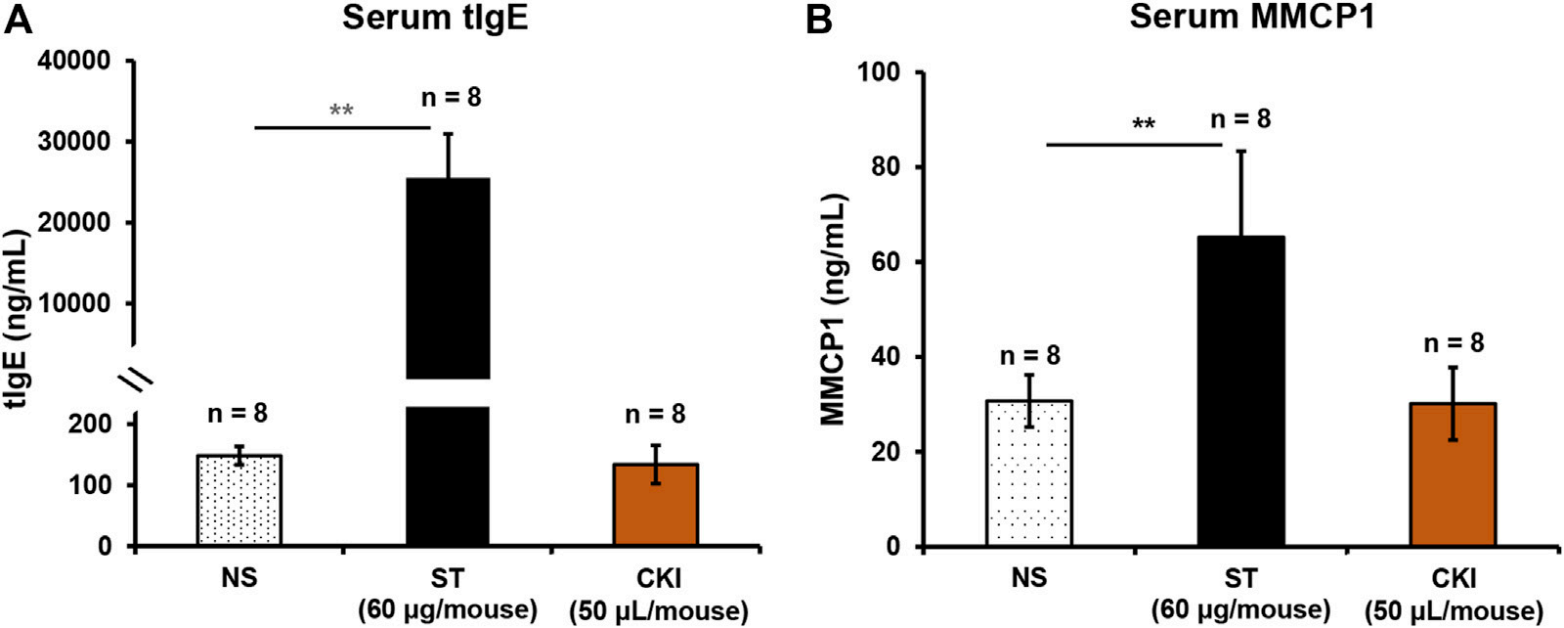
CKI-induced IHR is independent of IgE/FcεRI-mediated signaling. Mice were intraperitoneally injected with ST (60 µg/mouse) or CKI (50 µl/mouse) in the presence of aluminum adjuvant (100 µl/mouse) once a week. Seven days after the fifth immunization, serum tIgE and MMCP1 were determined using their respective commercial ELISA kits. ST was used as a positive control. **p < 0.01. CKI, Compound Kushen Injection; IHR, immediate hypersensitivity reaction; MMCP1, mast cell protease 1; NS, normal saline; ST, shrimp tropomyosin; tIgE, total IgE.

Clinical data (January 1996 - February 2020) showed that approximately 70% of CKI-induced ADRs occurred within 30 min after injection, belonging to typical IHRs (Dian et al., 2020). In contrast to IgG/FcγRIII-mediated IHR, IgE/FcεRI-mediated IHR is more common because it is easier to be triggered (MacGlashan, 2012; Finkelman et al., 2016; Montañez et al., 2017). If CKI-induced IHR is through IgE/FcεRI signaling, serum tIgE and MMCP1 (another specific marker for IgE-mediated mast cell activation (Khodoun et al., 2011)) levels should be significantly increased. We immunized the mice for 5 weeks by using a mixture of CKI and aluminum adjuvant (Gao et al., 2018). As a result, the positive control ST markedly elevated serum tIgE and MMCP1 levels after continuous intraperitoneal immunization, while CKI could not increase these two markers (Figure 2), demonstrating that CKI-induced IHR was independent of IgE/FcεRI-mediated signaling.

### CKI Is Able to Cause NIN-IHRs

**FIGURE 3:**
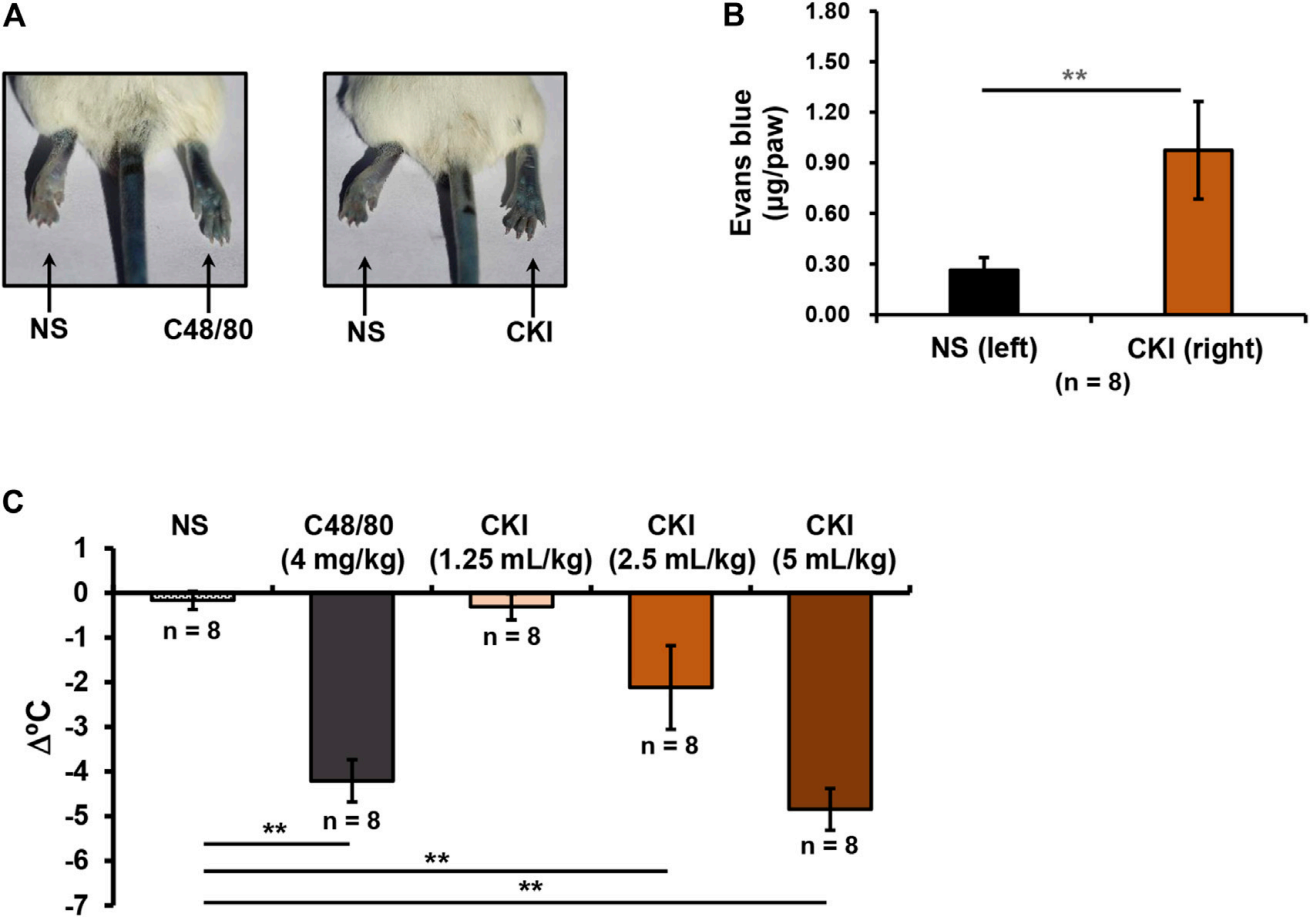
CKI can induce NIN-IHRs. **(A)** Representative images of Evans blue extravasation. Mice were injected (i.v.) with Evans blue (0.65 μmol). Five minutes later, right paw of mice was intraplantarly injected with CKI (10 μl/paw), and the left paw was injected with 10 μl of NS. C48/80 (0.3 μg/paw) was used as a positive control. Thirty minutes later, the mice were euthanized and photographed. **(B)** Quantification of Evans blue leakage into the paw. Paw tissues were collected and Evans blue in the paw tissues was extracted by DMF at 50°C overnight (>20 h). OD values were read at 620 nm. The concentration of the dye in the paw tissue was calculated according to the standard curve of Evans blue. **p < 0.01. **(C)** CKI induced hypothermia in propranolol-pretreated mice. The mice were pretreated (i.v.) with propranolol (0.118 μmol/mouse) 20 min before CKI (1.25–5 ml/kg) challenge (i.p.). C48/80 (4 mg/kg) was used as a positive control. The mice in the negative control group were received equivoluminal NS. Thirty minutes later, the rectal temperature was measured. **p < 0.01. C48/80, compound 48/80; CKI, Compound Kushen Injection; NIN-IHRs, non-immunologic immediate hypersensitivity reactions; NS, normal saline; OD, optical density.

In addition to IgE/FcεRI-mediated IHR, NIN-IHR is another important type of IHRs. In contrast to IN-IHRs, NIN-IHRs can be triggered after a single exposure to stimulus without the participation of antibody. To evaluate whether CKI could cause NIN-IHRs, local and systemic *in vivo* models were used. As shown in Figures 3A,B, whether positive control C48/80 (0.3 μg/paw) or CKI (10 μl/paw) significantly induced Evans blue extravasation of mouse paw after a single intraplantar injection. We next evaluated whether CKI could cause anaphylactoid shock (detected as hypothermia). To increase the sensitivity, mice were pretreated with propranolol which does not induce anaphylaxis by itself (TenBrook et al., 2004; Khodoun et al., 2009). As shown in Figure 3C, C48/80 led to a marked decrease of rectal temperature, and CKI (2.5 ml/kg - 5 ml/kg) also caused obvious hypothermia in the propranolol-pretreated mice. These results demonstrate that CKI can induce NIN-IHRs.

### PAF Receptor Antagonists Counter CKI-Induced NIN-IHR

**FIGURE 4:**
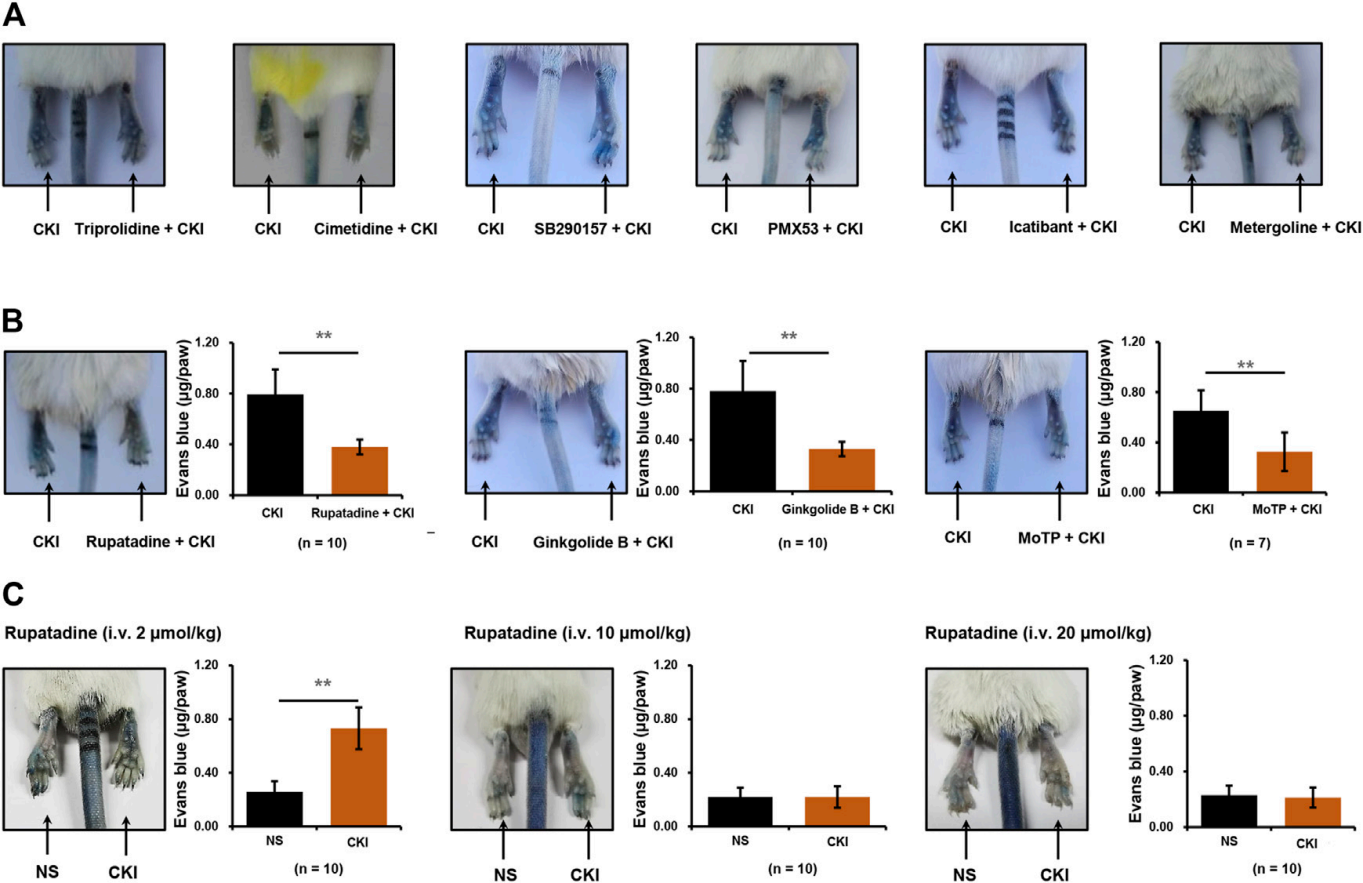
CKI-caused Evans blue leakage can be blocked by PAF receptor antagonists **(A)** Representative images of CKI-induced Evans blue extravasation. Right paws of mice were intraplantarly injected with 10 μl of different antagonists (60 mM triprolidine or 40 mM cimetidine or 3.8 mM SB290157 or 2 mM PMX53 or 1 mM icatibant or 12.4 mM metergoline), and the left paws were injected with 10 μl of NS. Ten minutes later, the mice were injected (i.v.) with Evans blue (0.65 μmol). Five minutes later, two paws were intraplantarly injected with CKI (10 μl/paw). Thirty minutes later, the mice were euthanized and photographed. **(B)** Representative images of Evans blue extravasation and quantification of Evans blue leakage in the paws with or without intraplantarly injecting PAF receptor antagonists (2 mM rupatadine or 2 mM ginkgolide B or 15 mM MoTP, 10 μl/paw). **p < 0.01. **(C)** Representative images of Evans blue extravasation and quantification of Evans blue leakage in the paws with or without intravenously injecting PAF receptor antagonist. The mice were pretreated (i.v.) with rupatadine (2–20 μmol/kg) 30 min before Evans blue injection (0.65 μmol). Five minutes after Evans blue injection, right paws of mice were intraplantarly injected with CKI (10 μl/paw), and the left paws were injected with 10 μl of NS. Thirty minutes later, the mice were euthanized. Paw tissues were collected and Evans blue in the paw tissues was extracted by DMF at 50°C overnight (>20 h). OD values were read at 620 nm. The concentration of the dye in the paw tissue was calculated according to the standard curve of Evans blue. **p < 0.01. CKI, Compound Kushen Injection; NS, normal saline; OD, optical density; PAF, platelet-activating factor.

NIN-IHR can be triggered by many mediators, such as histamine, complement-derived anaphylatoxin 3 or 5 (C3 or C5), bradykinin, 5-hydroxytryptamine and PAF, etc. (Vliagoftis et al., 1990; Muñoz-Cano et al., 2016). To unveil the underlying mechanism for CKI-induced NIN-IHR, a series of antagonists were used one by one. The obtained results showed that CKI-caused Evans blue leakage was not blocked by histamine H1/2 receptor antagonists (triprolidine and cimetidine), C3 or C5 antagonist (SB290157 and PMX53), bradykinin B2 receptor antagonist (icatibant) and 5-hydroxytryptamine receptor antagonist (metergoline) (Figure 4A). Finally, intraplantar injection of three PAF receptor antagonists (rupatadine, ginkgolide B and MoTP) significantly countered CKI-caused Evans blue leakage (Figure 4B). To further confirm the above results, the effect of systemic administration of PAF receptor antagonist was observed. As expectedly, except for the lowest dosage (2 μmol/kg), the other two dosages (10–20 μmol/kg) of rupatadine completely antagonized CKI-caused Evans blue leakage (Figure 4C). These results indicate that CKI-caused NIN-IHR is mediated by PAF.

### CKI Promotes PAF Production in Multiple Cells

**FIGURE 5:**
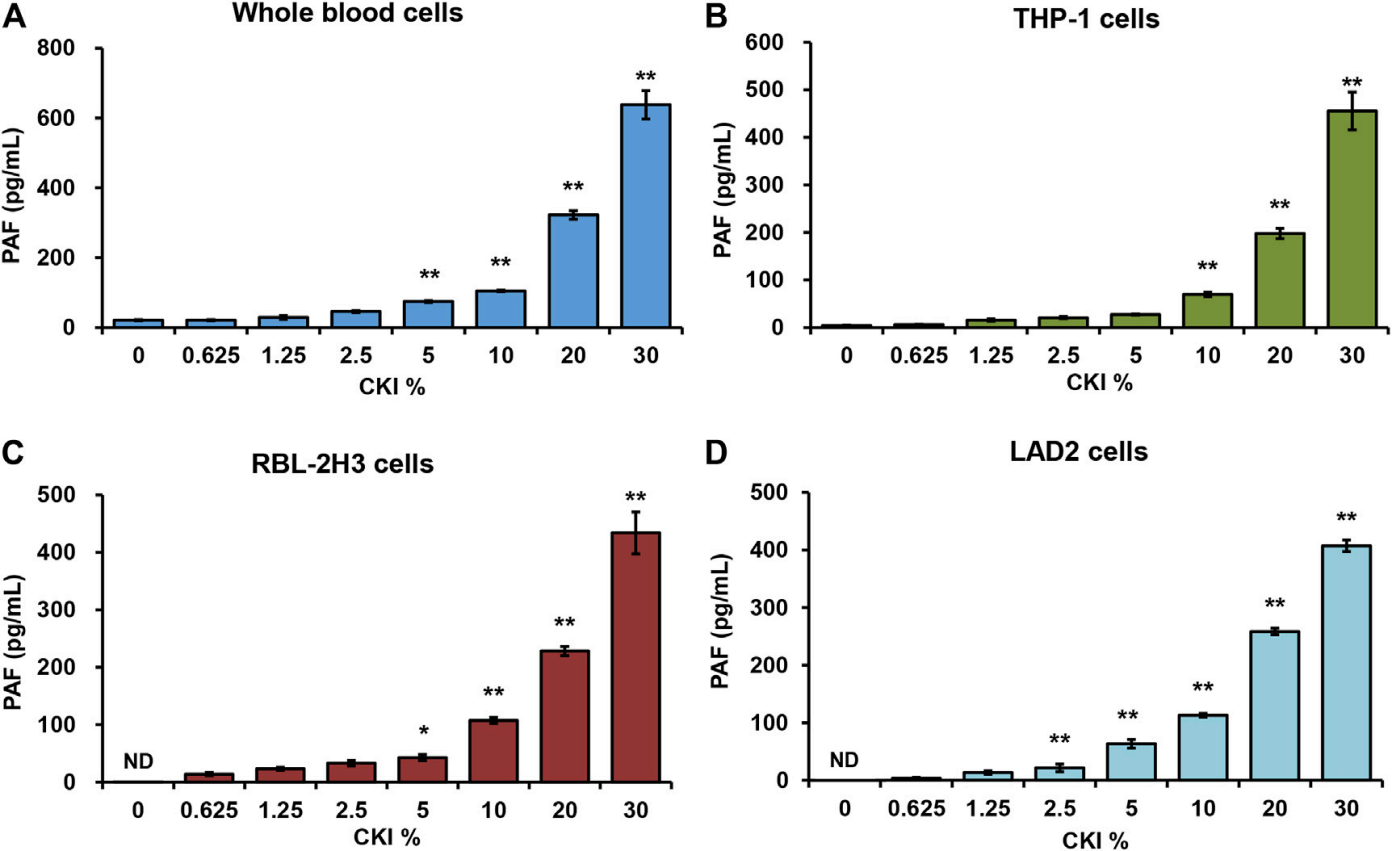
CKI promotes PAF production in **(A)** whole blood cells **(B)** THP-1 cells **(C)** RBL-2H3 cells and **(D)** LAD2 cells. Cells were incubated with CKI at 37°C for 1 h. The cell culture supernatant was collected and centrifuged (1,000 × g) at 4°C for 20 min. The supernatant was used for PAF assay by using commercial PAF ELISA kit. PAF concentration was calculated according to the standard curve. ^*^ *p* < 0.05 and **p < 0.01 *vs* 0% CKI. CKI, Compound Kushen Injection; ND, not detected; PAF, platelet-activating factor.

PAF is a short half-life but highly potent phospholipid which can be synthesized by a variety of cells (e.g., platelets, monocytes, neutrophils, basophils, and mast cells, etc.) in response to various stimuli (Chao and Olson, 1993; Gill et al., 2015). Given that CKI is an intravenous preparation, we first evaluated the effect of CKI on whole blood cells. As shown in Figure 5A, CKI was able to stimulate whole blood cells to release PAF in a concentration-dependent manner. Next, monocytes (THP-1), basophils (RBL-2H3) and mast cells (LAD2) were also subjected to CKI stimulation. Similarly, CKI could also promote PAF production in these three cells (Figures 5B–D), showing that its effect was non-cell-selective. Since RBL-2H3 cell line is more convenient to be cultured, we chose it in the subsequent active constituent and mechanism studies.

### HY-free CKI but Not SF-free CKI Contributes to NIN-IHRs

**FIGURE 6:**
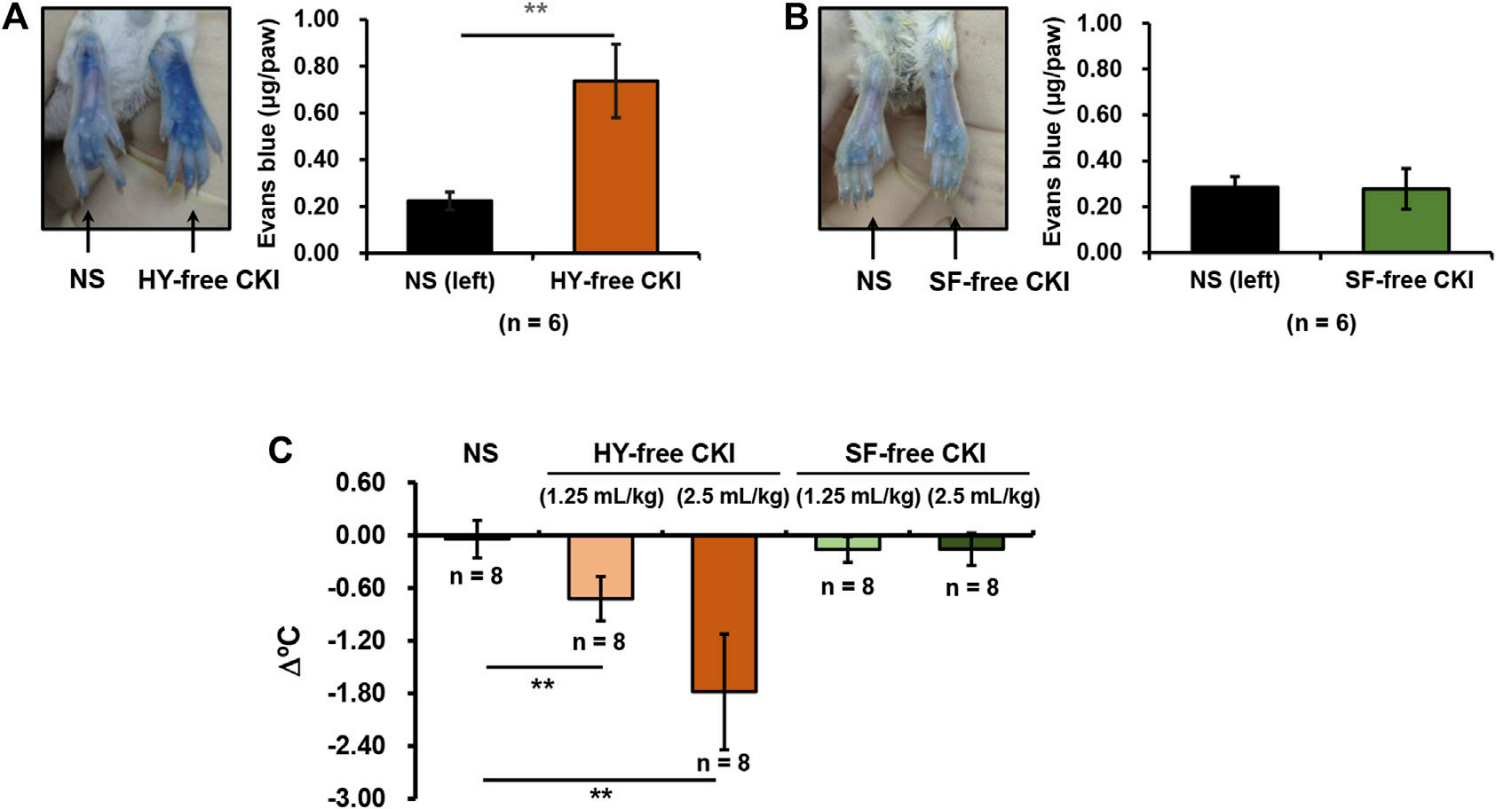
HY-free CKI but not SF-free CKI contributes to NIN-IHRs. **(A-B)** HY-free CKI was able to cause Evans blue leakage in the mouse paw. Mice were injected (i.v.) with Evans blue (0.65 μmol). Five minutes later, right paw of mice was intraplantarly injected with **(A)** HY-free CKI or **(B)** SF-free CKI (10 μl/paw), and the left paw was injected with equivoluminal NS. Thirty minutes later, the mice were euthanized and photographed. Paw tissues were collected and Evans blue in the paw tissues was extracted by DMF at 50°C overnight (>20 h). OD values were read at 620 nm. The concentration of the dye in the paw tissue was calculated according to the standard curve of Evans blue. **p < 0.01. **(C)** HY-free CKI was able to cause hypothermia in propranolol-pretreated mice. The mice were pretreated (i.v.) with propranolol (0.118 μmol/mouse). Twenty minutes later, the mice were intraperitoneally injected with HY-free CKI or SF-free CKI (1.25 ml/kg - 2.5 ml/kg). The mice in the negative control group were received equivoluminal NS. Thirty minutes later, the rectal temperature was measured. **p < 0.01. CKI, Compound Kushen Injection; HY, *Heterosmilax yunnanensis* Gagnep.; NIN-IHRs, non-immunologic immediate hypersensitivity reactions; NS, normal saline; OD, optical density; SF, *Sophora flavescens* Ait.

CKI is a *bis*-herbal formulation. To identify which one is the prime culprit for CKI-induced NIN-IHRs, we prepared HY-free CKI and SF-free CKI. The obtained data showed that HY-free CKI, rather than SF-free CKI, markedly caused Evans blue leakage in the paw after the first intraplantar injection (Figures 6A,B). Consistently, in the anaphylactoid shock model, HY-free CKI was able to significantly lower mouse rectal temperature (Figure 6C). These findings indicate that SF but not HY contributes to NIN-IHRs.

### SF-Alkaloids Are Responsible for CKI-Induced NIN-IHRs

**FIGURE 7:**
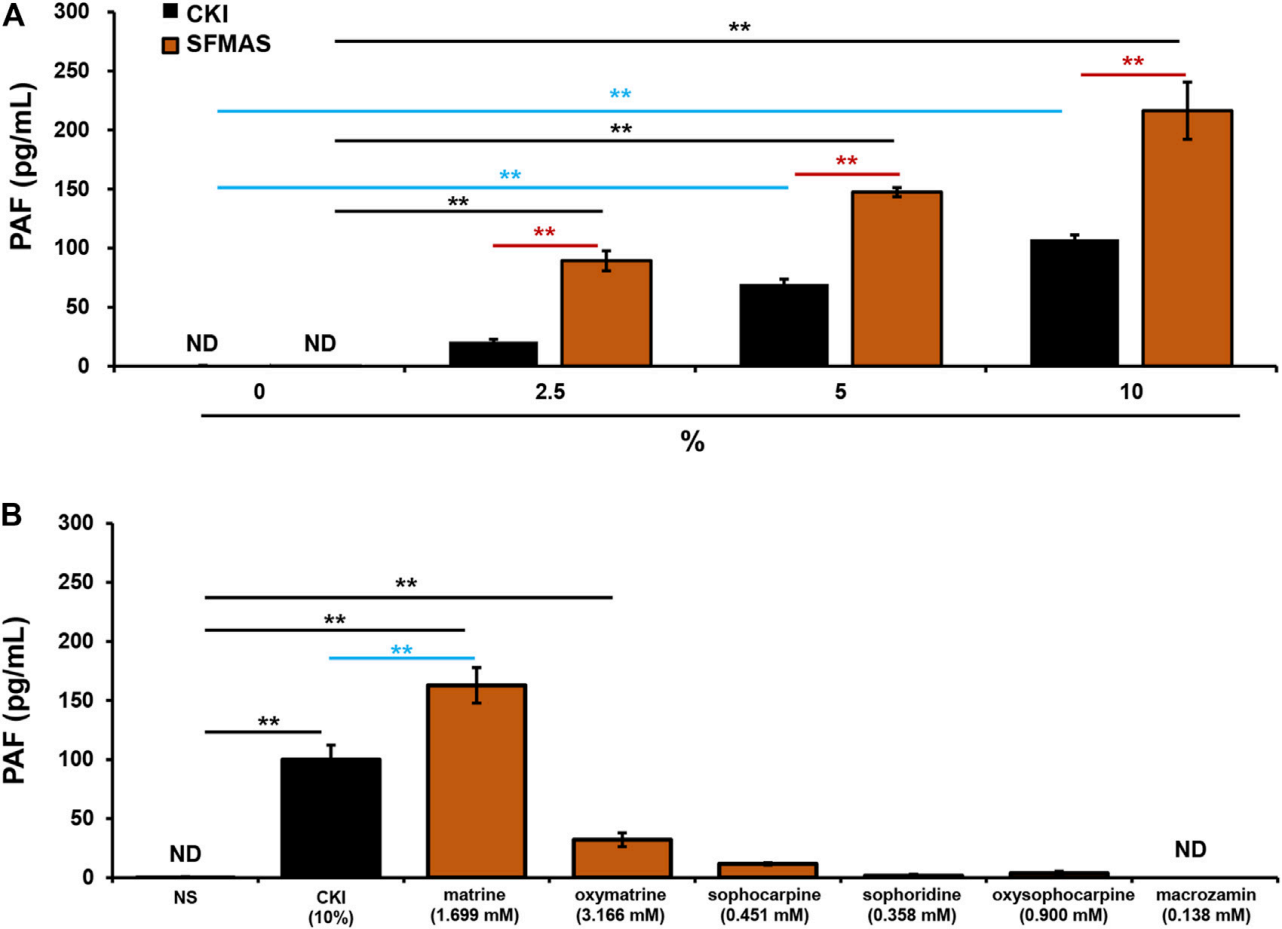
SF-alkaloids are responsible for CKI-induced NIN-IHRs. **(A)** Effects of CKI and SFMAS on PAF production in RBL-2H3 cells. RBL-2H3 cells were incubated with CKI or SFMAS at 37°C for 1 h. The cell culture supernatant was collected and centrifuged (1,000 × g) at 4°C for 20 min. The supernatant was used for PAF assay by using commercial PAF ELISA kit. **p < 0.01. **(B)** Effects of six quantified constituents in SF on PAF production in RBL-2H3 cells. RBL-2H3 cells were incubated with different constituents (1.699 mM matrine, 3.166 mM oxymatrine, 0.451 mM sophocarpine, 0.358 mM sophoridine, 0.900 mM oxysophocarpine, 0.138 mM macrozamin) at 37°C for 1 h. The cell culture supernatant was collected and centrifuged (1,000 × g) at 4°C for 20 min. The supernatant was used for PAF assay by using commercial PAF ELISA kit. **p < 0.01. CKI, Compound Kushen Injection; ND, not detected; NIN-IHRs, non-immunologic immediate hypersensitivity reactions; NS, normal saline; PAF, platelet-activating factor; SFMAS, *Sophora flavescens* Ait. main alkaloids solution.

The above results have demonstrated that SF was responsible for CKI-induced IHRs (Figure 6) and SF-alkaloids were the main constituents in CKI (Table 2). Thus, we prepared SF main alkaloids solution (SFMAS) through mixing five major alkaloids (matrine, oxymatrine, sophocarpine, sophoridine and oxysophocarpine) according to their respective proportions in CKI, and then compared its effect with CKI at equivalent concentrations. Unexpectedly, SFMAS exerted a stronger effect on PAF production (Figure 7A). To evaluate the contribution of six quantified constituents (Table 2) to IHR, we next compared their effects (at their respective concentrations equivalent to 10% CKI) with CKI. As a result, matrine and oxymatrine could significantly promote PAF production (Figure 7B). More unexpectedly, the capacity of matrine, the second abundant alkaloid in CKI, to promote PAF production even exceeded that of CKI (Figure 7B). These results strongly suggest that counteractive substances may exist in CKI, and SF-alkaloids are the prime culprits for CKI-induced NIN-IHRs.

### CKI or Matrine Promotes PAF Production via *de Novo* Pathway

**FIGURE 8:**
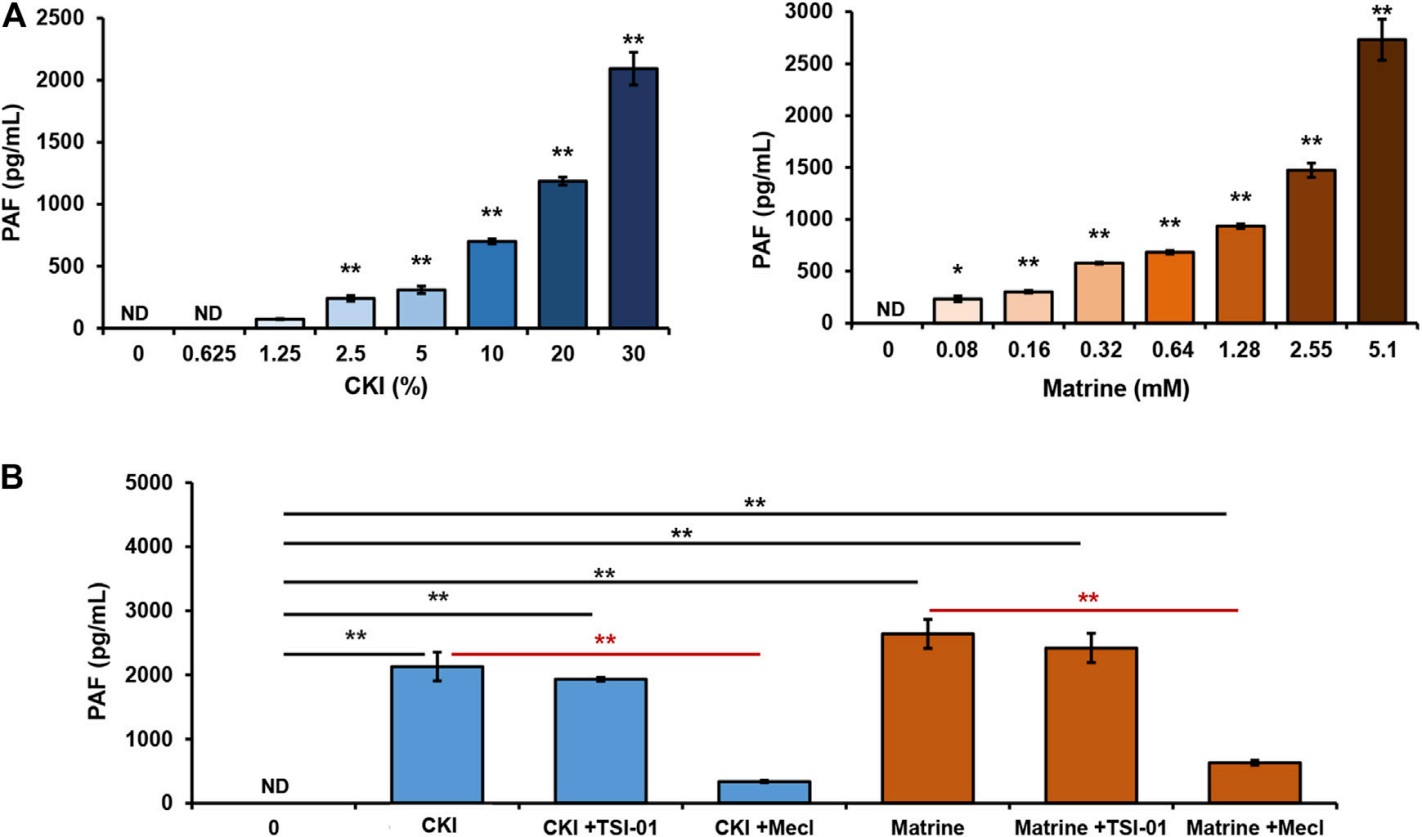
CKI or matrine promotes PAF production via *de novo* pathway. **(A)** Effect of CKI or matrine on PAF production in RBL-2H3 cell lysates. RBL-2H3 cells were lysed on ice by ultrasonication and the obtained cell lysates were centrifuged (5,000 × g) at 4°C for 5 min. The supernatant (85 μg protein) was incubated with CKI or matrine at 37°C for 1 h and then was centrifuged (1,000 × g) at 4°C for 20 min. PAF levels in the supernatant were assayed by using commercial PAF ELISA kit. PAF concentration was calculated according to the standard curve. ^*^ *p* < 0.05 and **p < 0.01 *vs* 0 group. **(B)** Mecl was able to counteract the effects of CKI and matrine on PAF production. RBL-2H3 cell lysates were incubated with CKI or matrine at 37°C for 1 h in the presence or absence of blocker (5 μM of TSI-01 or 20 μM of Mecl). Then PAF levels were assayed. **p < 0.01. CKI, Compound Kushen Injection; Mecl, meclofenoxate; ND, not detected.

PAF is produced through rapid synthesis in response to stimuli and is not stored intracellularly (Gill et al., 2015). Our previous finding also demonstrated that there was undetectable PAF inside RBL-2H3 cells (data not shown). An important question that arose was whether CKI-caused PAF production depended on the transmembrane signal. To address this issue, we determined the effect of CKI on PAF production in RBL-2H3 cell lysates. As a result, when the transmembrane signal pathway was cut off, the effect of CKI or matrine was still present, or rather stronger (Figure 8A), suggesting that CKI or matrine may directly activate certain synthetase(s) of PAF. To our knowledge, PAF can be synthesized via two pathways, remodeling pathway or *de novo* pathway (Gill et al., 2015). We next investigated which one could be activated by CKI or matrine. As shown in Figure 8B, the blocker of *de novo* pathway (Mecl), rather than remodeling pathway’s (TSI-01), could counteract the effects of CKI and matrine, indicating that they were through activating *de novo* pathway to promote PAF production.

## Discussion

PAF, also known as 1-0-alkyl-2-acetyl-*sn*-glycero-3-phosphocholine, is a highly potent phospholipid that plays an important role in the cause of numerous immune and inflammatory conditions (Venable et al., 1993). In the 1970s, PAF was first reported by a French immunologist, Jacques Benveniste, who demonstrated its relationship with histamine through an IgE-dependent process and as a mediator of anaphylaxis (a severe, rapid and life-threatening IHR) (Benveniste et al., 1972; Benveniste, 1974; Benveniste et al., 1977). Thereafter, the roles of PAF in anaphylaxis were successively elucidated. In murine models, PAF released by basophils plays a pivotal role in IgG-mediated anaphylaxis (Tsujimura et al., 2008). In humans with acute allergic reactions, the severity of anaphylaxis is positively correlated with serum PAF level while negatively correlated with PAF acetylhydrolase (PAF-AH) activity. Moreover, PAF-AH activity is the lowest in the patients with fatal anaphylactic reactions (Vadas et al., 2008). In our study, considering that serum levels of tIgE and MMCP1 were not significantly elevated in the CKI-immunized mice (Figure 2), there seemed to be a very low probability of CKI-induced PAF production through immunologic pathways.

PAF production can also be non-immunologically triggered by not only endogenic mediators (e.g., thrombin, histamine, ATP, IL-1 and TNF, etc.), but also ectogenic stimuli (e.g., A23187/PMA, zymosan and carbachol, etc.) (Chao and Olson, 1993). In fact, it was through promoting PAF production from multiple cells (Figure 5) that CKI leaded to NIN-IHRs (Figure 3). Various PAF receptor antagonists could significantly counter CKI-induced NIN-IHRs locally or systemically (Figures 4B,C).

PAF, a short half-life molecular, can be produced by many cell types in response to different stimuli and rapidly released outside cells (Chao and Olson, 1993; Gill et al., 2015). Our data showed that the effect of CKI on PAF production still existed in RBL-2H3 cell lysates (Figure 8A), indicating that this action was independent of transmembrane or intracellular signaling. Moreover, in contrast to cell systems (Figure 5), CKI exerted a stronger effect on PAF production in cell lysates (Figure 8A). This differentiation might be attributed to more sufficient interaction between CKI and its target enzyme(s).

PAF can be synthesized via two pathways, the remodeling pathway and the *de novo* pathway (Maclennan et al., 1996). The synthesis of PAF through remodeling pathway requires the conversion of 1-alkyl-2-acyl-*sn*-glycero-3-phosphocholine into lyso-PAF (the immediate precursor of PAF) which can be transferred to PAF by a specific acetyl-coenzyme A: lyso-PAF acetyltransferase. Alternatively, the *de novo* pathway begins with 1-alkyl-2-lyso-*sn*-glycero-3-phosphate which can be converted to PAF through enzyme cascades. In this study, we used TSI-01 (the inhibitor of lyso-PAF acetyltransferase in remodeling pathway) and Mecl (the inhibitor of 1-alkyl-2-acetyl-*sn*-glycerol cholinephosphotransferase in *de novo* pathway) for blocking these two pathways, respectively. As a result, TSI-01 had little impact on CKI-caused PAF production, whereas Mecl nearly abolished this effect of CKI (Figure 8B), suggesting that CKI might directly activate certain PAF-synthetase in *de novo* pathway.

To searching for the prime culprit for CKI-induced NIN-IHRs, we deliberately prepared HY-free CKI and SF-free CKI. As a result, HY-free CKI markedly caused Evans blue leakage and lowered rectal temperature (Figure 6). Further study showed that SFMAS exerted a stronger effect on PAF production than CKI at equivalent concentrations (Figure 7A). More unexpectedly, the capacity of matrine, the second abundant alkaloid in CKI, to promote PAF production also exceeded that of CKI (Figure 7B). These findings demonstrate that SF-alkaloids, especially matrine, are the prime culprits for CKI-induced NIN-IHRs.

Clinically, not all patients subjected to CKI suffer from IHRs, while the severity of attacks is also different. This situation is due to the existing opposing forces in producing- and degrading-PAF. The outcome appears when one of them is stronger than the other. Based on our findings, the main active constituents responsible for CKI-induced NIN-IHRs were alkaloids of SF (Figure 7). In humans, PAF-AH is mainly responsible for degrading PAF (Vadas et al., 2008). CKI-induced IHRs are more likely to occur in the patients with low activity of PAF-AH. Therefore, respectively monitoring the activity of PAF-AH in blood and the content of alkaloids, especially matrine in CKI, should be an effective strategy for predicting CKI-induced IHRs.

In summary, our study identifies, for the first time, that CKI can induce NIN-IHRs, rather than IgE-dependent IHRs, by promoting PAF production in a non-cell-selective manner. Whether local or systemic treatment of PAF receptor antagonists can counteract CKI-caused hypothermia or vascular leakage, which provides a potential strategy for treating CKI-induced IHRs. In the present study, we have unveiled that matrine is a potent PAF inducer, and CKI, a traditional Chinese medicine injection containing matrine at mM level, induces NIN-IHRs via the *de novo* pathway. However, it is still unclear which one(s) is activated in this enzyme cascade. Further studies are needed to clarify more exact mechanisms for the activation of matrine or CKI on enzyme(s).

## Data Availability

The original contributions presented in the study are included in the article/Supplementary Material, further inquiries can be directed to the corresponding authors.

## Abbreviations

ADRs, adverse drug reactions; C48/80, compound 48/80; CKI, Compound Kushen Injection; HY, Heterosmilax yunnanensis Gagnep; IHRs, immediate hypersensitivity reactions; IN-IHRs, immunologic immediate hypersensitivity reactions; Mecl, meclofenoxate; MMCP1, mouse mast cell protease 1; ND, not detected; NIN-IHRs, non-immunologic immediate hypersensitivity reaction; NS, normal saline; OD, optical density; PAF, platelet-activating factor; PAF-AH, platelet-activating factor acetylhydrolase; SF, Sophora flavescens Ait; SFMAS, Sophora flavescens Ait. main alkaloids solution; ST, shrimp tropomyosin; tIgE, total IgE.
