# Supplementary material for: Compound Kushen Injection Induces Immediate Hypersensitivity Reaction Through Promoting the Production of Platelet-Activating Factor via de Novo Pathway
Source: Front Pharmacol. 2021 Oct 8;12:768643. doi: 10.3389/fphar.2021.768643 (PMC8531113; doi:10.3389/fphar.2021.768643)
Supplement: Supplementary file 7 [file DataSheet1.DOCX]

**CKI:**


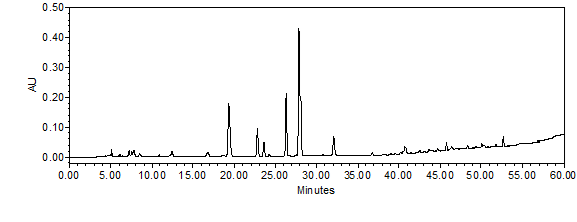


8

7

6

5

4

3

2

1

**HY-free CKI:**


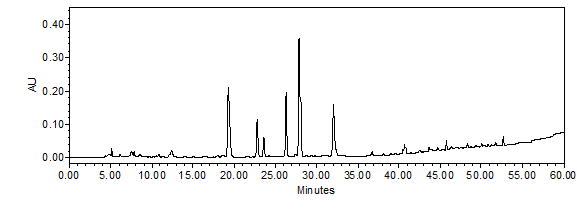


8

7

6

5

4

3

2

**SF-free CKI:**


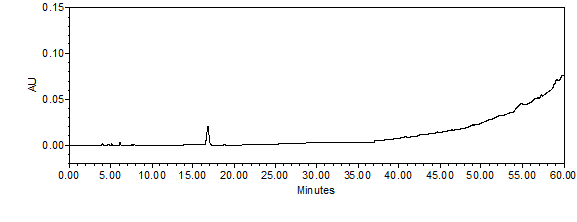


1

| Sample | Test values | macrozamin | matrine | sophocarpine | sophoridine | oxysophocarpine | oxymatrine |
| --- | --- | --- | --- | --- | --- | --- | --- |
| CKI  (Lot^#^ 20181034) | Peak area | 200676 | 2276908 | 835539 | 404935 | 1624042 | 3982978 |
|  | Con.(mg/mL) | 0.53 | 4.22 | 1.11 | 0.89 | 2.36 | 8.37 |
| SF-free CKI | Peak area | 392368 | / | / | / | / | / |
|  | Con.(mg/mL) | 1.072 | / | / | / | / | / |
| HY-free CKI | Peak area | / | 3779833 | 1393365 | 645004 | 2053013 | 4182753 |
|  | Con.(mg/mL) | / | 7.268 | 1.921 | 1.473 | 3.104 | 9.145 |
